# Influence of pH on bile sensitivity amongst various strains of *Listeria monocytogenes* under aerobic and anaerobic conditions

**DOI:** 10.1099/jmm.0.000160

**Published:** 2015-11

**Authors:** Sally J. White, Daniel M. McClung, Jessica G. Wilson, Brandy N. Roberts, Janet R. Donaldson

**Affiliations:** Department of Biological Sciences, Mississippi State University, Mississippi State, MS 39762, USA

## Abstract

*Listeria monocytogenes* is a dangerous bacterium that causes the food-borne disease listeriosis and accounts for nearly 20 % of food-borne deaths. This organism can survive the body's natural defences within the digestive tract, including acidic conditions and bile. Although the bile response has been analysed, limited information is available concerning the ability of *L. monocytogenes* to resist bile under anaerobic conditions, especially at acidic pH, which mimics conditions within the duodenum. Additionally, it is not known how the bile response varies between serotypes. In this study, the survival of strains representing six serotypes was analysed under aerobic and anaerobic conditions following exposure to bile. Exposure to bile salts at acidic pH increased toxicity of bile, resulting in a significant reduction in survival for all strains tested. However, following this initial reduction, no significant reduction was observed for an additional 2 h except for strain 10403S (*P* = 0.002). Anaerobic cultivation increased bile resistance, but a significant increase was only observed in virulent strains when exposed to bile at pH 5.5. Exposure to pH 3.0 prior to bile decreased viability amongst avirulent strains in bile in acidic conditions; oxygen availability did not influence viability. Together, the data suggested that being able to sense and respond to oxygen availability may influence the expression of stress response mechanisms, and this response may correspond to disease outcome. Further research is needed on additional strains to determine how *L. monocytogenes* senses and responds to oxygen and how this varies between invasive and non-invasive strains.

## Introduction

*Listeria monocytogenes* is a Gram-positive, food-borne pathogen that is responsible for nearly 20 % of food-related deaths in the USA (Scallan *et al.*, 2011). This microbe causes the opportunistic infection listeriosis and primarily affects pregnant women, the elderly and the immunocompromised (Farber & Peterkin, 1991). Upon ingestion, *L. monocytogenes* must survive a variety of stressors, including acidic conditions of the stomach, bile encountered within the gastrointestinal tract and hypoxic/anoxic conditions. Being able to efficiently respond to these stressors is key for survival within the host.

Following exposure to the acidic conditions within the stomach, *L. monocytogenes* is exposed to bile secreted into the duodenum by the liver during digestion (Monte *et al.*, 2009). This complex fluid is composed primarily of bile acids, cholesterol, phospholipids and bilirubin (Coleman *et al.*, 1979). Bile acids are the bactericidal component of bile, being able to disrupt the cell membrane and DNA (Bernstein *et al.*, 1999; Coleman *et al.*, 1979; Prieto *et al.*, 2004, 2006). The primary bile acids (cholic acid and chenodeoxycholic acid) are formed from cholesterol in the liver and are subsequently conjugated with either a glycine or taurine prior to being secreted into the small intestine. Secondary bile acids (deoxycholic acid and lithocholic acid) can form due to dehydroxylation reactions performed by gut microbes (Merritt & Donaldson, 2009). Peptide linkages between secondary bile acids and glycine or taurine also form conjugated bile acids, such as glycodeoxycholic acid (GDCA) and taurodeoxycholic acid (TDCA). Human bile is primarily composed of these conjugated bile acids (Shioda *et al.*, 1969).

The toxicity of bile is dependent upon pH (Begley *et al.*, 2002). For example, the *L. monocytogenes* strain EGDe (serotype 1/2a) had decreased survival in *ex vivo* bile at pH 5.5 in comparison with pH 7. Survival under these acidic conditions was found to be dependent upon the sigma stress factor (*sig* ^B^), principal virulence factor (*prfA*), bile salt hydrolase (*bsh*) and bile exclusion system (*bilE*). These resistance mechanisms, however, were not necessary for survival in bile at pH 7, which would be encountered within the gall bladder (Dowd *et al.*, 2011). Hypoxic conditions have been linked to increased expression of *bsh*, which may influence bile resistance under anaerobic conditions (Dussurget *et al.*, 2002). However, the *L. monocytogenes* strain LO28 (serotype 1/2c) had increased sensitivity to bile acids at pH 5.5 in comparison with pH 7.5 when cultured under anaerobic conditions (Begley *et al.*, 2002). These data suggest that availability of oxygen may influence bile resistance, but the impact on survival may vary between strains.

Although much is known concerning the response of *L. monocytogenes* to stressors encountered within the gastrointestinal tract, little is understood in regard to these responses in anaerobic conditions, especially with respect to variations between serotypes. Therefore, the goal of this study was to examine the effect of pH on bile toxicity of different serotypes in the presence or absence of oxygen.

## Methods

### Bacterial strains

Bacterial strains used in this study are listed in Table 1. Frozen stocks were stored at − 80 °C and grown on Tryptic Soy Agar (TSA) prior to cultivation in Tryptic Soy Broth (TSB) at 37 °C.

**Table 1.** Strains used in this study

Strain	Serotype	Isolation source
F2365	4b	Human – epidemic
EGDe	1/2a	Animal – sporadic
10403S	1/2a	Human – sporadic
15313	1/2a	Animal – sporadic
HCC23	4a	Catfish
HCC7	1	Catfish
2011L-2663	1/2b	Human – cantaloupe outbreak
2011L-2676	1/2a	Human – cantaloupe outbreak
ScottA	4b	Human – epidemic
LO28	1/2c	Human – sporadic

### Survival analysis in bile

Overnight cultures were inoculated into fresh TSB and allowed to grow to mid exponential phase (OD_600_ 0.35–0.4) at 37 °C, at which point cells were pelleted (8000 ***g*** for 5 min) and resuspended in fresh media at pH 7.5 or 5.5 supplemented with either 0 or 1 % porcine bile extract (Sigma). Aliquots (0.1 ml) were removed at 0, 1, 2 and 3 h post-exposure to bile extract, serially diluted in PBS, and plated onto TSA. Plates were incubated at 37 °C for 16 h prior to enumeration. A minimum of three independent replicates were performed. The log_10_c.f.u. ml^− 1^ values were calculated and the mean determined amongst the replicates. Percent viability was assessed based on non-bile-treated samples for each pH condition tested. GraphPad Prism was used to analyse the differences between pH on growth using an unpaired *t*-test, with significance declared at *P* < 0.05. For correlation analysis, survival was first normalized against the starting concentration at time 0, then compared with growth over time between strains using Pearson's correlation (xlstat version 2015 1.01; Addinsoft), with *P* < 0.05 indicating significance.

### pH dependency on bile extract, GDCA and TDCA toxicity

Isolates from freshly streaked cultures were used to inoculate 5 ml TSB for cultivation at 16 h at 37 °C. This culture was added to fresh media (1 % inoculum) with 1 % porcine bile extract (Sigma), 5 mM GDCA (Sigma) or 5 mM TDCA (Sigma) at pH 7.5, 6.5, 5.5 or 4.5, adjusted with 10 N HCl where needed. Cultures were incubated for 16 h at 37 °C, at which time aliquots (0.1 ml) were serially diluted in PBS, plated onto TSA and incubated for 16 h prior to enumeration. The log_10_c.f.u. ml^− 1^ values were calculated and the mean determined amongst three independent replicates. Percent survival was assessed based on non-bile-treated samples for each pH condition tested. Prism (GraphPad) was used to analyse the differences between pH on growth within each treatment using an unpaired *t*-test, with *P* < 0.05 declared as significant.

### Anaerobic cultivation

Media to be used for anaerobic cultivation were prepared at least 2 days prior to use. Briefly, TSB was adjusted to pH 7.5, 6.5, 5.5 or 4.5, autoclaved and immediately placed in a Coy Anaerobic Airlock chamber (gas mix 95 % N_2_/5 % H_2_). Anaerobic conditions were monitored throughout the duration of the study with an oxygen monitor and also with a resazurin control sample.

Isolates from freshly streaked cultures were used to inoculate 5 ml TSB for cultivation at 16 h at 37 °C. Overnight cultures (1 %) were used to inoculate the TSB previously prepared and then were subsequently supplemented with either 0 or 1 % porcine bile extract (Sigma). Cultures were incubated at 37 °C within the anaerobic chamber for 16 h, after which aliquots (0.1 ml) were removed, serially diluted in PBS and plated onto TSA. Plates were incubated anaerobically for 24 h at 37 °C. Per cent survival was determined for each replication based on TSB media only controls. Prism (GraphPad) was used to analyse the differences between the survival of strains in bile under aerobic or anaerobic conditions using an unpaired *t*-test, with *P* < 0.05 declared as significant. At least three independent replicates were analysed.

### Acid cross-protection to bile assay

Overnight cultures were diluted 1 : 100 in fresh TSB and allowed to incubate to late exponential phase (OD_600_ 0.8), at which time media were exchanged for fresh TSB at pH 7.5 or 3.0. Cells were exposed to the acidic condition for 1 h, after which cells were pelleted, washed in PBS and resuspended in fresh TSB supplemented with either 0 or 1 % porcine bile extract at pH 7.5 or 5.5 for 16 h at 37 °C. Samples were serially diluted in PBS and plated onto TSA. Experiments analysed under anaerobic conditions were performed exactly as described for aerobic conditions, with the exception of incubation in a Coy anaerobic chamber (gas mix 95 % N_2_/5 % H_2_). Per cent survival was calculated based on the viability of controls not treated with bile extract, with respect to appropriate pH treatment. Prism (GraphPad) was used to analyse the impact of pretreatment with acid on bile survival using an unpaired *t*-test, with *P* < 0.05 declared as significant. A minimum of three independent replicates were analysed.

## Results and Discussion

### Survival of *L. monocytogenes* in bile

The toxicity of bile is directly related to pH (Begley *et al.*, 2002). However, it is not known whether the severity of this bactericidal effect is similar amongst different serotypes of *L. monocytogenes.* Variation has been observed in bile (King *et al.*, 2003; Merritt *et al.*, 2010; Payne *et al.*, 2013) and acid (King *et al.*, 2003; Koutsoumanis *et al.*, 2003; Liu *et al.*, 2005; Melo *et al.*, 2013; Olesen *et al.*, 2009) resistance between different strains of *L. monocytogenes.* Due to this variation, it is imperative to analyse the biological response utilizing a multi-strain approach. *Listeria monocytogenes* consists of four genetic lineages (Orsi *et al.*, 2011). Strains used in this study were chosen based on serotype and genetic lineage, as well as isolation source. Six of the 13 serotypes are represented in the current study. Genetic lineage I strains tested were F2365, ScottA and 2011L-2663. Genetic lineage II strains tested were EGDe, 10403S, 15313, 2011L-2676 and LO28. These two genetic lineages represent serotypes primarily associated with human cases of listeriosis and 95 % of the characterized isolates collected from recent outbreaks (1/2a, 1/2b and 4b), although variations exist in their pathogenic potential. For instance, F2365 (serotype 4b), 2011L-2663 (serotype 1/2b) and 2011L-2676 (serotype 1/2a) were isolated from two of the deadliest listeriosis outbreaks in history (Laksanalamai *et al.*, 2012; Linnan *et al.*, 1988). However, 15313 is avirulent in a mouse model, although it is a serotype 1/2a strain that contains genes related to virulence (Erdenlig *et al.*, 2000; Kathariou & Pine, 1991; Liu *et al.*, 2003). The 10403S strain also has reduced virulence in comparison with EGDe (Bécavin *et al.*, 2014). Genetic lineage III and IV isolates are rare and primarily associated with animals; HCC23 and HCC7 were chosen to represent these lineages. The evolutionary divergence between these serotypes has been described by others (Ragon *et al.*, 2008; Rychli *et al.*, 2014). Although many genomic comparisons have been conducted to decipher the virulent genome, limited information is known about the physiological response of these different strains to stressors encountered within the human gastrointestinal tract. Even within lineages, some genes can be lost, modifying the ability of some strains to adapt to certain environments. For instance, proteomic analysis supports that bile salt tolerance varies amongst strains and regulation of stress response differs between serotypes (Payne *et al.*, 2013). Additionally, genetic comparisons and evolution of the bacteria have been proposed to result in a loss of virulence (den Bakker *et al.*, 2010; Ragon *et al.*, 2008). Therefore, it is imperative to analyse multiple strains to gain a better understanding of how invasive strains and non-invasive strains respond to the host's environment and how this influences the outcome of disease. However, it is noted that this study is still very limited in terms of strain selection, as only 10 strains were analysed.

**Fig. 1.**
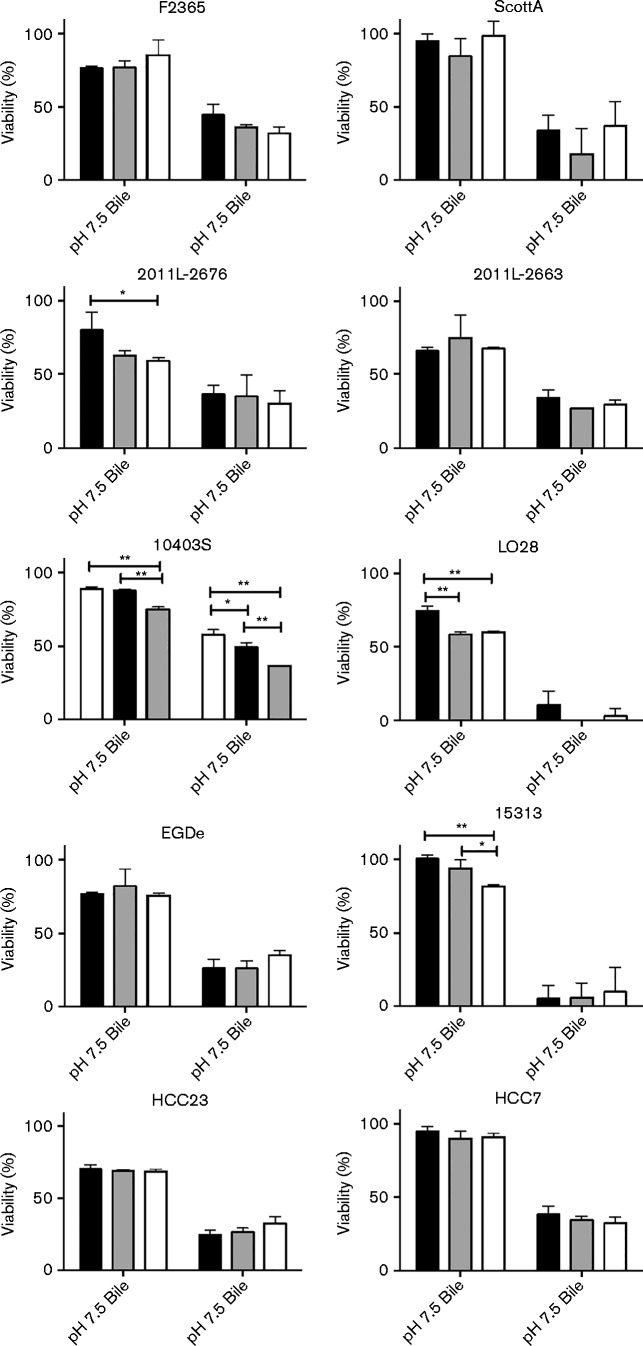
Viability of *L. monocytogenes* in porcine bile extract at pH 7.5 and 5.5. *L. monocytogenes* F2365, ScottA, 2011L-2636, 2011L-2676, 10403S, 15313, HCC23, HCC7, LO28 and EGDe were grown to mid exponential phase prior to treatment for 3 h in the following conditions: TSB pH 7.5 with either 0 or 1 % bile, or TSB pH 5.5 with either 0 or 1 % bile. Percent viability was calculated at 1 (black), 2 (grey) and 3 h (white) post-exposure to bile in relation to TSB-only controls at the respective pH. Results represent mean ± sd of three independent experiments. *P < 0.05; **P < 0.001.

Bile resistance is one of the key aspects to the infectious potential of *L. monocytogenes* (Gahan & Hill, 2014; Merritt & Donaldson, 2009). To determine the impact bile has on the growth of *L. monocytogenes* when in the more toxic environment of reduced pH, the survival of 10 different strains was determined hourly post-exposure to bile at pH 7.5 or 5.5 (Fig. 1). Viability was assessed at 1, 2 and 3 h post-exposure to bile; percent viability for each condition was determined based on the viable cell counts of non-bile-treated samples for individual time points. Exposure to 1 % bile at pH 7.5 decreased viability for 2011L-2676, 10403S, LO28 and 15313 during the 3 h time period analysed (*P* < 0.05). This was unexpected, as the other strains that represent genetic lineages I and II did not exhibit a significant decrease in survival under these conditions (*P*>0.05). In fact, F2365 showed a significant increase in viability within 3 h post-exposure to bile (*P* < 0.001).

Growth at pH 5.5 was only impacted for 2011L-2676 (*P* < 0.05). Bile at pH 5.5 increased toxicity against all strains tested (*P* < 0.005; Fig. 1). However, the addition of bile did not impact survival significantly over the 3 h time course in comparison with TSB (pH 5.5)-only controls, indicating that the impact on viability was primarily due to the reduction in pH. In fact, only 10403S exhibited a decrease in survival following exposure to bile at pH 5.5 over the 3 h time course analysed (*P* < 0.05). Viability was not impacted for F2365, ScottA, 2011L-2676, 2011L-2663, LO28, EGDe, 15313, HCC23 and HCC7 (Fig. 1). Interestingly, ScottA, 15313, EGDe and HCC23 showed a slight increase in growth following exposure to bile, although this increase was not significant (Fig. 1). This suggests that 10403S has an impaired ability to efficiently respond to bile in acidic conditions.

The survival exhibited by certain strains of *L. monocytogenes* following exposure to bile at acidic pH could indicate the emergence of acid-resistant subpopulations that has been noted by others (Metselaar *et al.*, 2013). Resistant subpopulations have been noted for LO28 following exposure to pH 3.5. Therefore, it is possible that the viability observed following exposure of mid-exponential-phase cells to bile at pH 5.5 was due to selected acid-resistant populations. Additional data are needed to analyse the impact of these subpopulations on extended exposure to bile.

Analysis of the viable bacterial populations following exposure to bile at reduced pH normalized against the concentration prior to exposure indicated that a correlation existed between the survival of ScottA with 2011L-2676 (*P* = 0.044) and HCC23 with EGDe (*P* = 0.049). Although these strains represent two different genetic lineages, this suggests that they may utilize a similar response mechanism in the presence of bile. As ScottA and EGDe have been found to have different glutamate decarboxylase activities, the impact of pH on bile resistance may be attributed to variations in acid resistance mechanisms (Olier *et al.*, 2004).

### Sensitivity of *L. monocytogenes* to TDCA and GDCA

**Fig. 2.**
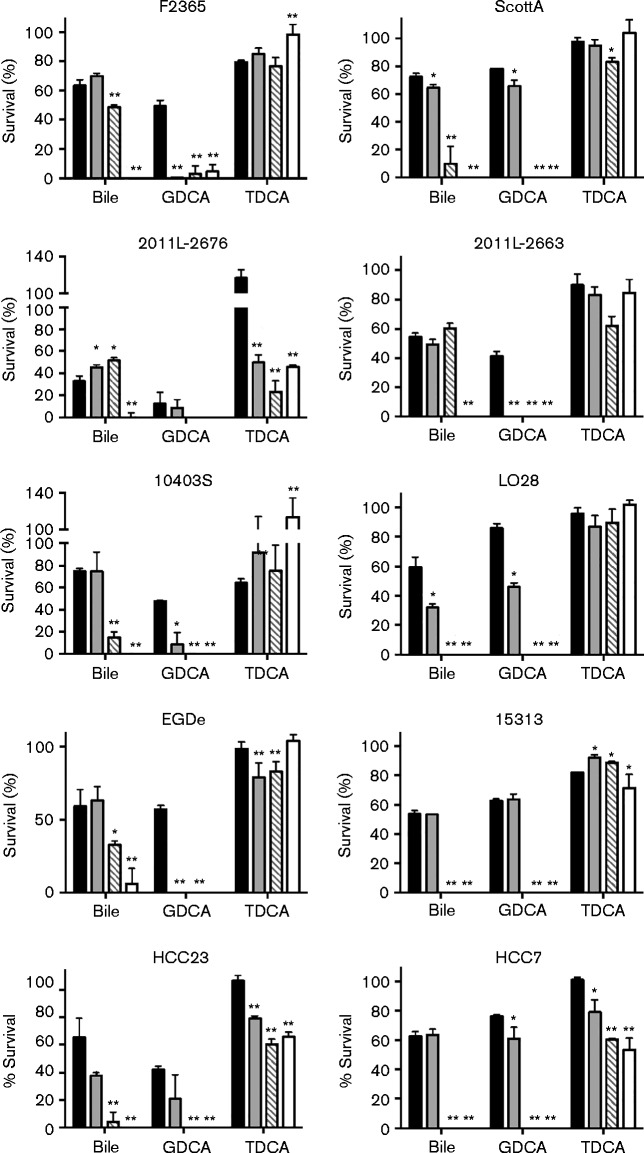
Influence of pH on toxicity of GDCA and TDCA. *L. monocytogenes* F2365, ScottA, 2011L-2636, 2011L-2676, 10403S, 15313, HCC23, HCC7, LO28 and EGDe were cultured in TSB at pH 7.5 (black), 6.5 (grey), 5.5 (hatched) or 4.5 (white). Cultures were grown for 16 h in media supplemented with 1 % porcine bile extract, 5 mM GDCA or 5 mM TDCA. Viability was assessed by plate counts and per cent survival was determined in relation to TSB-only controls at the respective pH. Results represent mean ± sd of three independent experiments. *P < 0.05; **P < 0.001.

Human bile is primarily composed of conjugated bile acids (Coleman *et al.*, 1979; Shioda *et al.*, 1969). Therefore, as variations were observed in survival in bile extract at reduced pH, the survival of these strains was analysed in media supplemented with the bile acids GDCA and TDCA at pH 7.5, 6.5, 5.5, or 4.5. GDCA at the concentration of 5 mM has been previously shown to be more toxic than 5 mM TDCA at reduced pH (Begley *et al.*, 2002; De Smet *et al.*, 1995). As expected, survival was severely impaired for all strains analysed after cultivation in media at pH 5.5 or 4.5 with GDCA for 16 h (Fig. 2); only F2365 showed detectable growth at pH 5.5 and 4.5. Bile acid toxicity under acidic conditions has been linked to cell death through the release of reactive oxygen species in eukaryotic cells (Jenkins *et al.*, 2007), but has not been assessed under these conditions in bacteria. The impact of GDCA toxicity on energy demand by *Listeria* needs to be assessed.

The toxic effect that TDCA had on survival of *L. monocytogenes* varied between strains (Fig. 2). A reduction in survival was only evident for 2011L-2676, 15313, HCC23 and HCC7 (*P* < 0.05). Although two of the four 1/2a strains tested showed a significant decrease in survival at pH 4.5 in comparison with the media-only control (2011L-2676 and 15313), all other strains without a significant difference belonged to genetic lineages I and II.

Extended exposure to bile extract at reduced pH severely impacted survival. All strains tested showed a significant decrease in viability at pH 4.5. Strains that were isolated from the 2011 cantaloupe outbreak actually showed an increase in bile resistance at pH 5.5 in comparison with neutral pH. Together, these data suggest that an additional component to the bile may influence the survival of *L. monocytogenes* or that the influence on survival is strain specific. It is possible that the mechanism utilized may differ between strains and that this response may influence the outcome of disease. Further research is needed to dissect these two possibilities.

### Anaerobic conditions increase survival of *L. monocytogenes* against bile salts at reduced pH

**Fig. 3.**
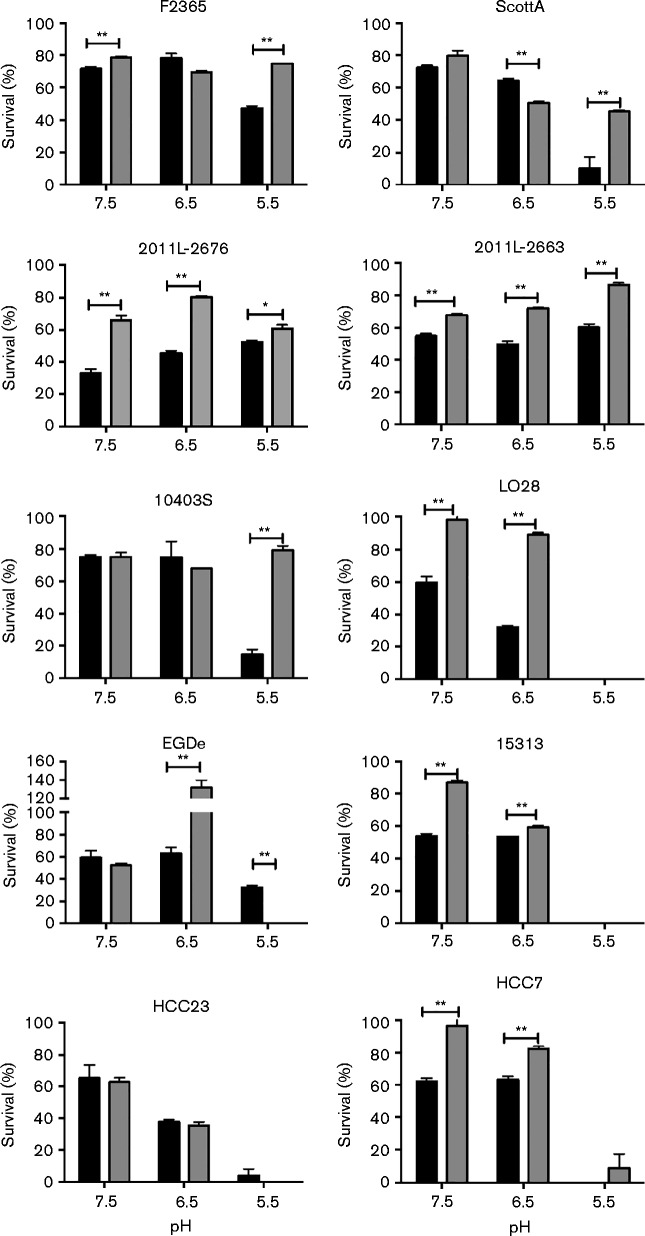
Influence of anaerobic cultivation on bile resistance of *L. monocytogenes*. *L. monocytogenes* F2365, ScottA, 2011L-2636, 2011L-2676, 10403S, 15313, HCC23, HCC7, LO28 and EGDe were cultured in media supplemented with either 0 or 1 % bile under either aerobic (black) or anaerobic (grey) conditions. Percent survivals were determined in relation to media-only controls at the respective pH. Results represent mean ± sd of three independent experiments. *P < 0.05; **P < 0.001.

A previous study indicated that the activity of bile salt hydrolase increases under anaerobic conditions (Dussurget *et al.*, 2002). As variations were observed in the survival of *L. monocytogenes* when exposed to bile at reduced pH, the influence of anaerobic conditions was tested on bile resistance. Strains representing various serotypes were analysed following 16 h cultivation in media supplemented with 1 % porcine bile extract at pH 7.5, 6.5, 5.5 or 4.5 (Fig. 3). At pH 7.5 under anaerobic conditions, F2365, 2011L-2663, 2011L-2676, LO28, 15313 and HCC7 showed a significant increase in survival in comparison with growth under aerobic conditions (*P* < 0.05). Contrary to previous studies, increased sensitivity to bile at pH 7.5 was not observed in EGDe in relation to other *Listeria* strains, particularly LO28 and ScottA (Begley *et al.*, 2002). This difference is most likely due to variations in the cultivation methods used in this study in comparison with the previous study.

Interestingly, strains isolated from epidemics of listeriosis all showed a significant increase in resistance at pH 5.5 under anaerobic conditions. F2365, ScottA, 2011L-2676, 2011L-2663 and 10403S showed an increase in survival at pH 5.5 in the presence of bile under anaerobic conditions (*P* < 0.05). This is very interesting to note, as this mimics the environment that *L. monocytogenes* would be exposed to when entering the duodenum. Survival in this part of the small intestine may allow for the upregulation of stress response mechanisms that will enhance invasiveness at deeper parts of the intestinal tract. This is in agreement with studies that have indicated that anaerobic cultivation increases the invasion potential of *L. monocytogenes* (Bo Andersen *et al.*, 2007). The only strains that showed growth following 16 h incubation in media at pH 4.5 with bile were 2011L-2676 and EGDe. Anaerobic conditions did not influence the survival of these strains at pH 4.5 (*P*>0.05; data not shown).

### Pre-exposure to acid does not improve bile survival of *L. monocytogenes*

**Fig. 4.**
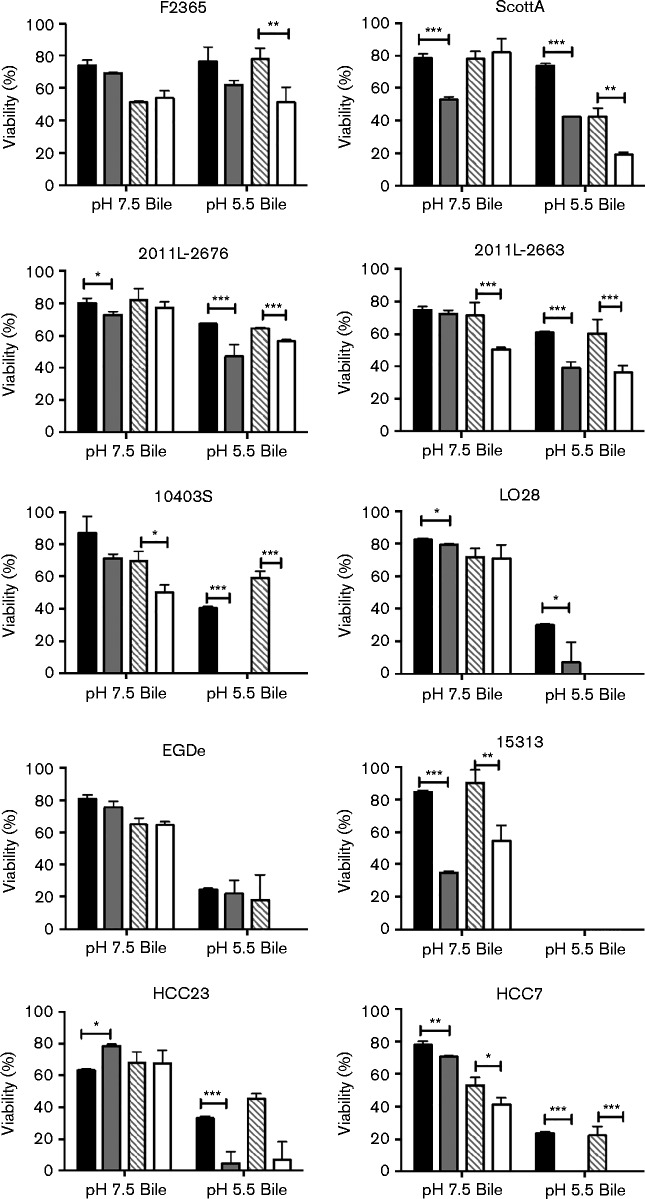
Viability of *L. monocytogenes* in bile when pre-exposed to acidic conditions. *L. monocytogenes* F2365, ScottA, 2011L-2636, 2011L-2676, 10403S, 15313, HCC23, HCC7, LO28 and EGDe were grown to late exponential phase, exposed to TSB at pH 7.5 or 3.0 for 1 h and then cultured in media at pH 7.5 or 5.5 supplemented with either 0 or 1 % bile for 16 h. Per cent survivals were determined in relation to media-only controls at the respective pH. Aerobic cultures exposed to pH 7.5 media, followed by exposure to bile (black); aerobic cultures exposed to pH 3.0 media, followed by exposure to bile (grey); anaerobic cultures exposed to pH 7.5 media, followed by exposure to bile (hatched); anaerobic cultures exposed to pH 3.0 media, followed by exposure to bile (white). Results represent mean ± sd of three independent experiments. *P < 0.05; **P < 0.001; ***P < 0.0001.

The first stressor that *L. monocytogenes* encounters following ingestion is the acidic condition of the stomach. In addition to the alteration in pH, this also marks the first point of transition to a hypoxic environment (He *et al.*, 1999). Although *L. monocytogenes* possesses acid tolerance mechanisms, the impact that exposure to pH 3.0 has on survival following exposure to additional stressors encountered within the gastrointestinal tract is not known, especially with regard to reduced oxygen availability. To determine if exposure to acidic pH 3.0, which closely mimics that of the stomach, can enhance survival under conditions encountered within the duodenum, various *L. monocytogenes* strains were exposed to media at pH 3.0 for 1 h, followed by exposure to media at pH 7.5 or 5.5 supplemented with bile extract. However, it should be noted that this analysis was performed with TSB at reduced pH, rather than in simulated gastric fluid. Fig. 4 represents the impact that pre-exposure to pH 3.0 has on survival of *L. monocytogenes* following exposure to media supplemented with bile. Survival was determined based on viability following exposure to bile in comparison with identical treatment without bile. Pretreatment to acidic conditions only increased the survival of HCC23 following exposure to bile at pH 7.5 under aerobic conditions (*P* < 0.001). For all other strains tested, exposure to the acidic conditions decreased the survival following exposure to bile at pH 7.5. Exposure to acidic conditions under anaerobic conditions did, ironically, stabilize viability of F2365, ScottA, 2011-2676, LO28, EGDe and HCC23 following exposure to bile at pH 7.5.

To determine if exposure to acidic conditions encountered within the stomach (pH 3.0) increased the ability of *L. monocytogenes* to survive conditions encountered in the duodenum (pH 5.5 and bile), viability was assessed following pretreatment with either pH 7.5 or 3.0 prior to treatment with bile at pH 5.5 under aerobic or anaerobic conditions (Fig. 4). None of the strains tested showed an increase in viability in bile when pretreated with media at pH 3.0. This indicates that exposure to acidic conditions does not improve viability of *L. monocytogenes* against stressors encountered in the gastrointestinal tract. In fact, exposure to acid prior to bile exposure increased the sensitivity of most strains analysed. Additionally, as there was no impact on survival of acid pretreated samples and non-treated samples based on oxygen availability, this provides further support for the involvement of oxygen availability, not acid exposure, in increased resistance against stressors. Tolerance to acidic conditions encountered within the stomach is important for initial survival, but does not seem to provide cross-protection against conditions encountered in the small intestine. However, it is possible that exposure to conditions more closely simulating *in vivo* conditions, such as simulated gastric fluid, may result in different results. This will need to be further analysed in future studies.

Together, these data suggest that the ability of *L. monocytogenes* to sense oxygen may influence stress resistance. However, the impact of oxygen availability on bile resistance was not consistent amongst all strains analysed. This could indicate that the expression of stress response mechanisms differs between strains. Although studies have been conducted to analyse the genomic comparisons between serotypes, this study indicates that there is a necessity to decipher the transcriptome of multiple strains in response to stressors encountered under physiologically relevant conditions. It is also imperative that additional strains are analysed in future studies to determine correlations to resistance amongst *L. monocytogenes.*
